# Supplementary material for: Independent Stage Classification for Gastroesophageal Junction Adenocarcinoma
Source: Cancers (Basel). 2023 Oct 25;15(21):5137. doi: 10.3390/cancers15215137 (PMC10650394; doi:10.3390/cancers15215137)

Supplemental Figure S1. Survival curve by gross ypTN group.

A. Upper and middle esophagus

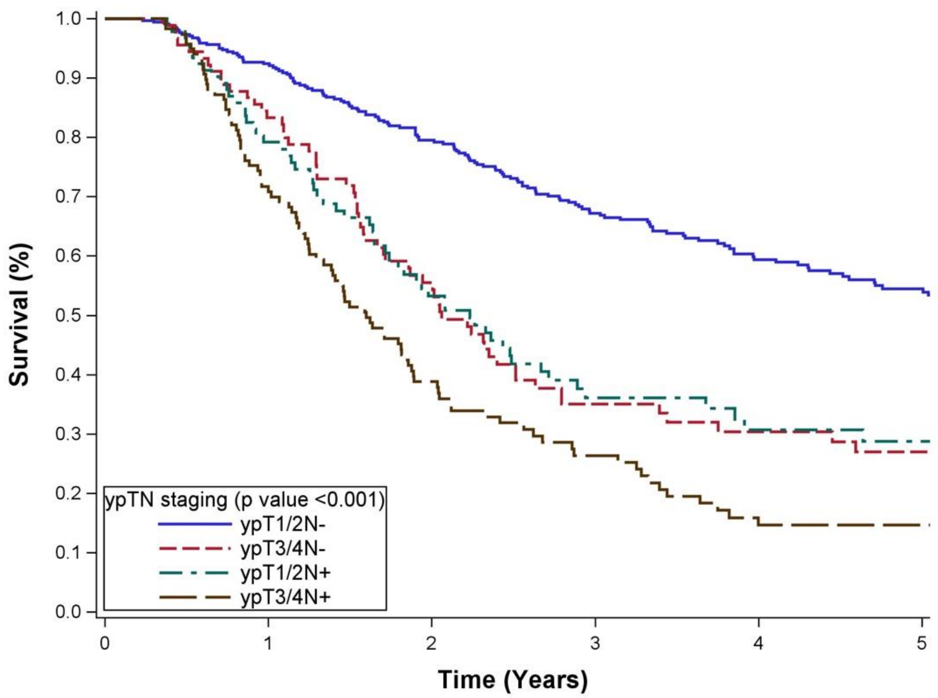

B. Lower esophagus

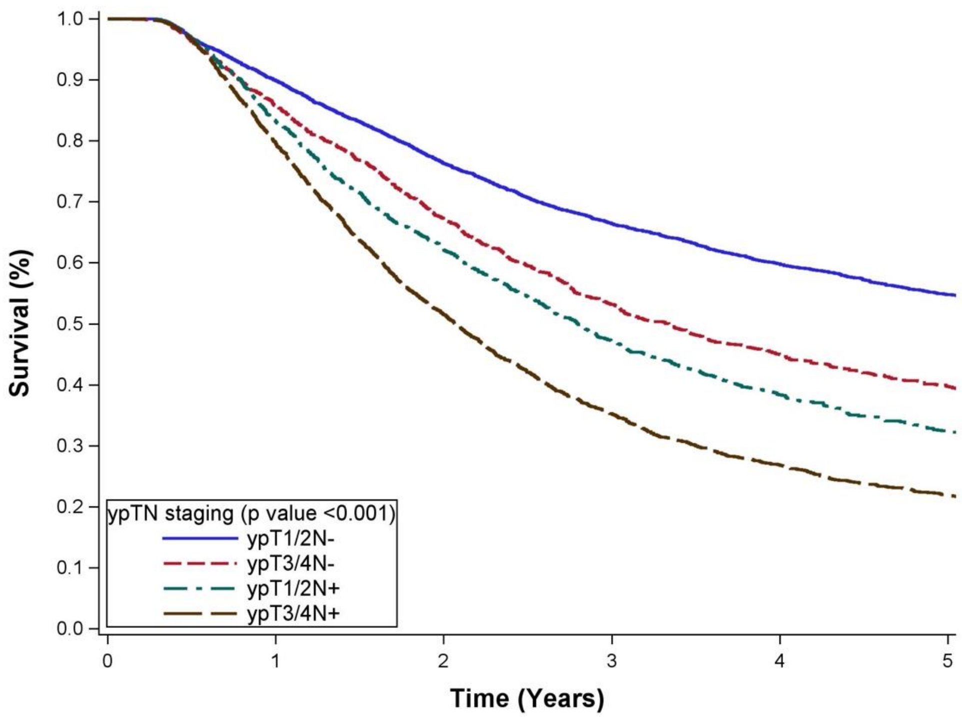

C. Gastric cardia

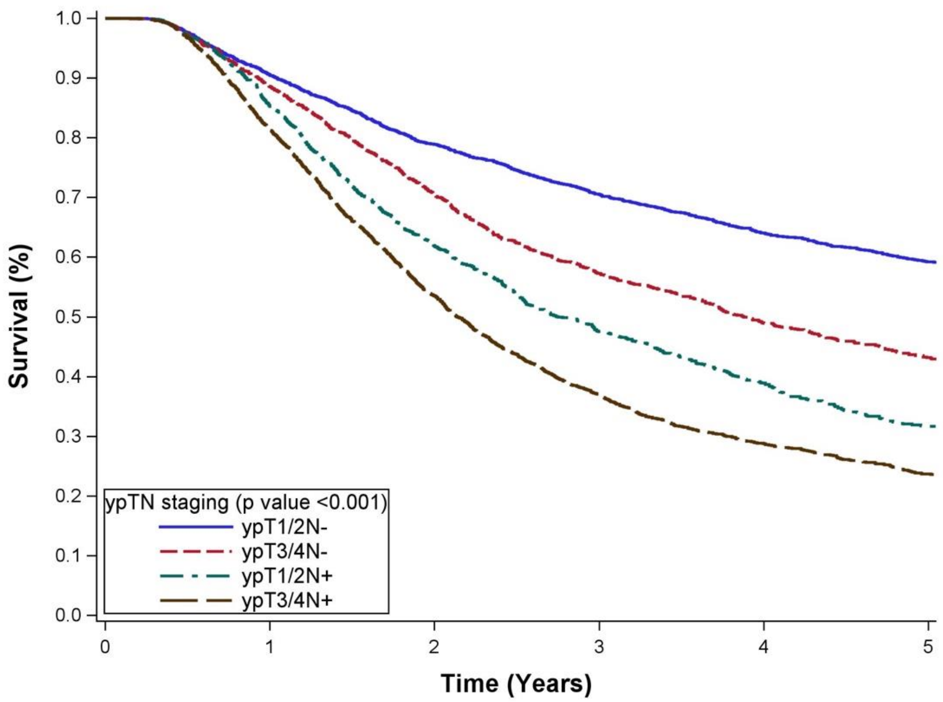

D. Other stomach

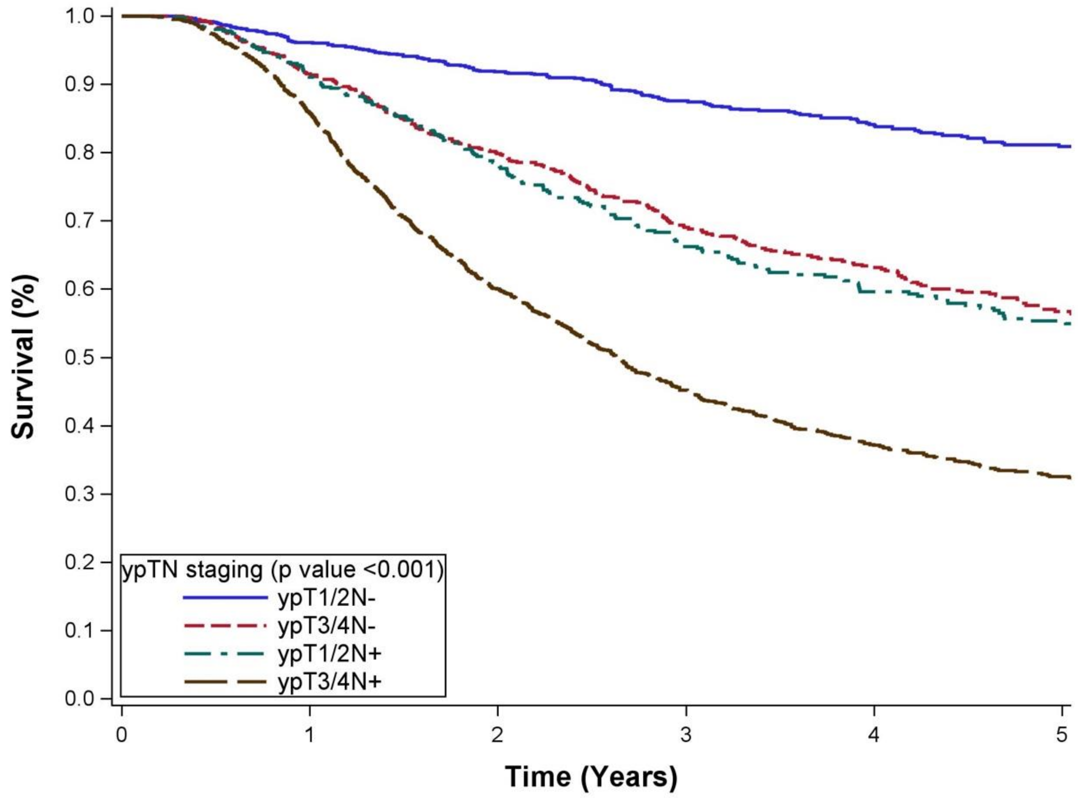

Supplement: Supplementary file 1 [file cancers-15-05137-s001.zip › cancers-2563243-supplementary.pdf]
